# A Novel Compound, “FA-1” Isolated from *Prunus mume*, Protects Human Bronchial Epithelial Cells and Keratinocytes from Cigarette Smoke Extract-Induced Damage

**DOI:** 10.1038/s41598-018-29701-2

**Published:** 2018-07-31

**Authors:** Andrew J. Jang, Ji-Hyeok Lee, Mari Yotsu-Yamashita, Joodong Park, Steve Kye, Raymond L. Benza, Michael J. Passineau, You-Jin Jeon, Toru Nyunoya

**Affiliations:** 1 Cardiovascular Institute, Department of Medicine, Allegheny Health Network, Pittsburgh, PA 15212 USA; 2 Lee Gil Ya Cancer and Diabetes Institute, 7-45, Songdodong, Yeonsugu, Incheon, 406-840 Republic of Korea; 3 Graduate School of Agricultural Science, Tohoku University, 468-1 Aramaki-Aza-Aoba, Aoba-ku, Sendai, Miyagi 980-0845 Japan; 4 Fysee Inc., 131, Angam-ro, Angseong-myeon, Chungju-si, Chungcheongbuk-do 27303 Republic of Korea; 5 Acerta Pharma, 2200 Bridge Parkway, Suite 101, Redwood City, CA 94065 USA; 6 Department of Marine Life Sciences, Jeju National University, Jeju, 690-756 Republic of Korea; 7 Department of Medicine, University of Pittsburgh, Pittsburgh, PA 15213 USA

## Abstract

Extract of the Japanese apricot (JAE) has biological properties as an antioxidant and anti-inflammatory agent. We hypothesized that JAE might exert therapeutic effects on cigarette smoke (CS)-induced DNA damage and cytotoxicity. In this study, we found that concentrated JAE protects against cigarette smoke extract (CSE)-induced cytotoxicity and DNA damage accompanied by increased levels of aldehyde dehydrogenase (ALDH)2, 3A1, and Werner’s syndrome protein (WRN) in immortalized human bronchial epithelial cells (HBEC2) and normal human epidermal keratinocytes (NHEK). Using the centrifugal partition chromatography (CPC) method, we identified an undescribed compound, 5-hydroxymethyl-2-furaldehyde bis(5-formylfurfuryl) acetal (which we named FA-1), responsible for the protective effects against CSE. This chemical structure has not been reported from a natural source to date. Protective effects of isolated FA-1 against CSE were observed in both HBEC2 and NHEK cells. The studies described herein suggest that FA-1 isolated from JAE protects against CSE-induced DNA damage and apoptosis by augmenting multiple isozymes of ALDH and DNA repair and reducing oxidative stress.

## Introduction

Japanese apricot (JA), *Prunus mume*, has long been popular as a health food among the South Korean, Japanese, and Chinese populations. Japanese apricot extract (JAE) has various beneficial biological effects, such as reducing oxidative stress and inflammation, and augmenting innate immunity^1–5^. For example, concentrated JAE may prevent cardiovascular disease as an anti-oxidative effect on angiotensin II-induced ROS generation^6^. In addition, triterpenoids in JAE have both anti-tumor and anti-inflammatory effects^7^. However, the potential utility of JAE to provide a positive effect in smoking-associated conditions, such as COPD and skin aging has not yet been evaluated.

Cigarette smoking (CS) is a major risk factor for chronic obstructive pulmonary disease (COPD)^8^. CS contains abundant toxic chemicals, including reactive aldehydes^9^ that cause DNA damage and cell death. Members of the aldehyde dehydrogenase (ALDH) superfamily, consisting of nineteen NAD(P)^+^-dependent isozymes in humans, catalyze aldehyde oxidation^10^. We recently demonstrated that CS exposure induces gene expression of multiple isozymes of ALDH in cultured immortalized human bronchial epithelial cells (HBEC2). ALDH3A1, the most robustly induced isozyme among the ALDH superfamily, attenuates CS-induced DNA damage and cytotoxicity^11^. Many different ALDH species, including ALDH 2 and 3A1, catalyze the oxidation of aliphatic, aromatic, and lipid peroxidation (LPO)-derived aldehydes, including 4-hydroxy-2-nonenal (4-HNE)^10^. ALDH3A1 also attenuate LPO-mediated growth inhibition^12^, UV light-induced cytotoxicity^13^, ROS-induced protein modification^14^, and genotoxin-induced DNA damage and apoptosis^11,15^.

CS activates the DNA damage response (DDR) that is mediated by phosphoinositide 3-kinase related protein kinases (PIKKs), including ataxia teleangiectasia mutated (ATM)^16,17^. Upon formation of DNA double-strand breaks (DSBs), ATM is activated by autophosphorylation at the serine 1981 residue and phosphorylates the serine 139 residue of H2AX variant (γH2AX) on chromatin flanking DSB sites. γH2AX is required to relay the subsequent DDR signaling and DNA repair^18–20^. We previously showed that Werner’s syndrome (WRN) protein, a RecQ helicase member, plays an important role in DNA repair in CS-induced DNA damage and cellular senescence *in vitro*^16^. Further, CS exposure enhances proteasome-dependent degradation of WRN protein that is detrimental as WRN protein is required to protect against CSE-induced DNA damage^16,21^.

In the present study, we successfully identified the novel molecule of 5-hydroxymethyl-2-furaldehyde bis(5-formylfurfuryl) acetal (which we named FA-1) isolated from concentrated JAE. Based upon our findings, FA-1 appears to be responsible for the protective effects of JAE on cytotoxicity, DNA damage, and oxidative stress in cigarette smoke extract (CSE)-exposed cells. These protective effects appear to be mediated by augmented ALDH and DNA repair.

## Results

### Concentrated JAE protects against CSE-induced cytotoxicity and increases ALDH and WRN protein in immortalized human bronchial epithelial cells

**Figure 1:**
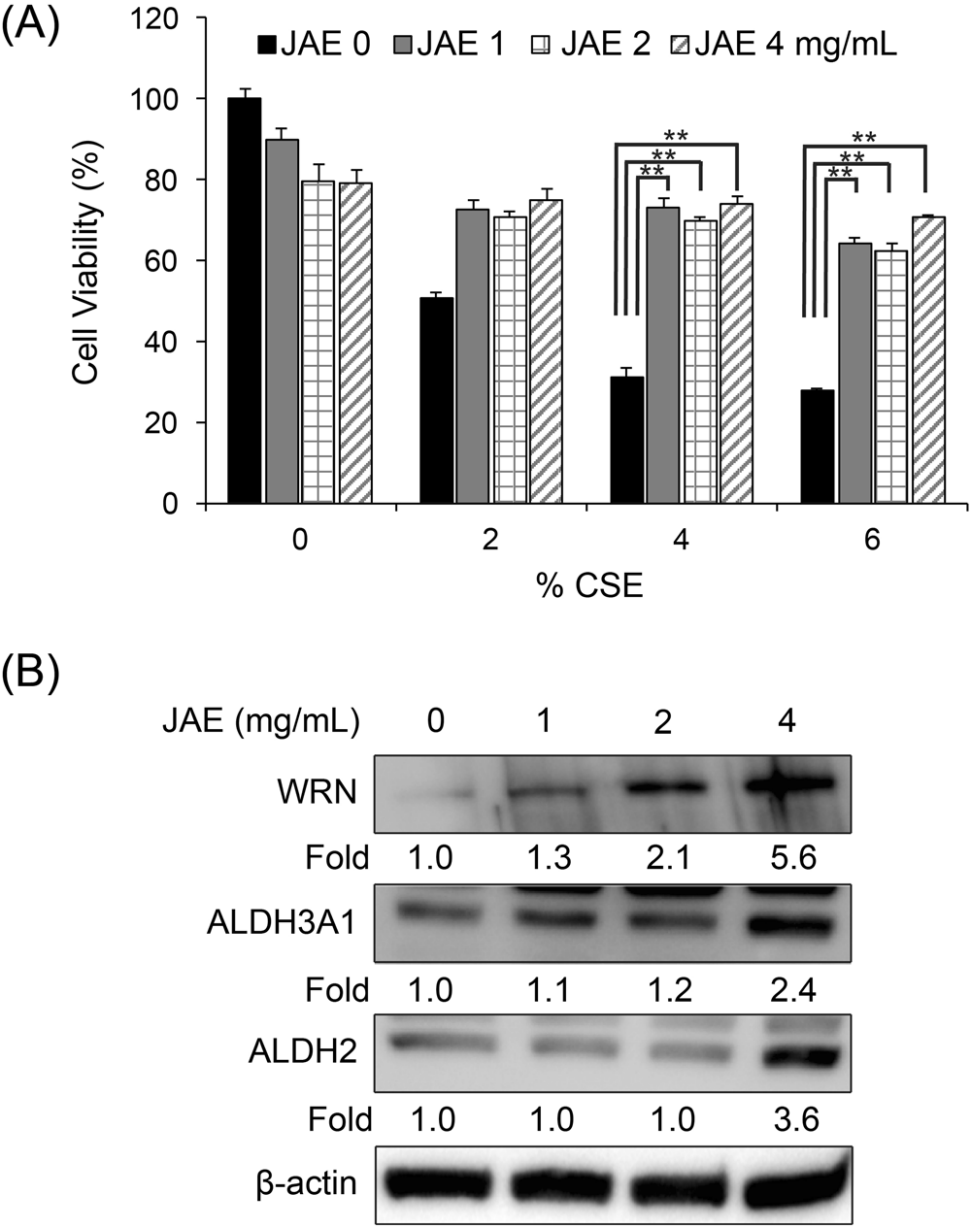
JAE increases WRN and ALDH proteins in immortalized human bronchoepithelial cells. (**A**) Immortalized HBEC2 cells were cultured in the absence or presence of CSE (0, 2, 4, and 6%) for 24 h after treatment with JAE (0, 1, 2, and 4 mg/mL) for 24 h. Cell viability was determined by the MTT assay. (**B**) Immortalized HBEC2 cells were cultured in absence or presence of 1.5% CSE for 24 h after treatment with JAE (0, 1, 2, and 4 mg/mL) for 24 h. Immunoblot analysis was performed for WRN, ALDH2, and ALDH3A1 protein. Data are expressed as mean ± SEM for two independent experiments with three triplicate samples.

To determine the effects of unpurified Japanese apricot extract (JAE) on CSE-induced cytotoxicity, HBEC2 cells were cultured with various concentrations (0, 1, 2, and 4 mg/mL) of JAE. JAE at all concentrations protected against CSE-induced cytotoxicity according to the MTT assay (Fig. 1A). Furthermore, we determined the effect of JAE on ALDH and WRN protein *in vitro*; HBEC2 cells were cultured with JAE (1, 2, and 4 mg/mL) or the vehicle (Corresponding to 1.0% volume of sterile H_2_O) and JAE enhanced ALDH2, ADLH3A1, and WRN protein expression in a dose dependent manner (Fig. 1B). These data suggest that JAE protects against CSE-induced cytotoxicity and enhances ALDH2, ALDH3A1 and WRN protein in HBEC2.

### Isolation and structural determination of FA-1

**Figure 2:**
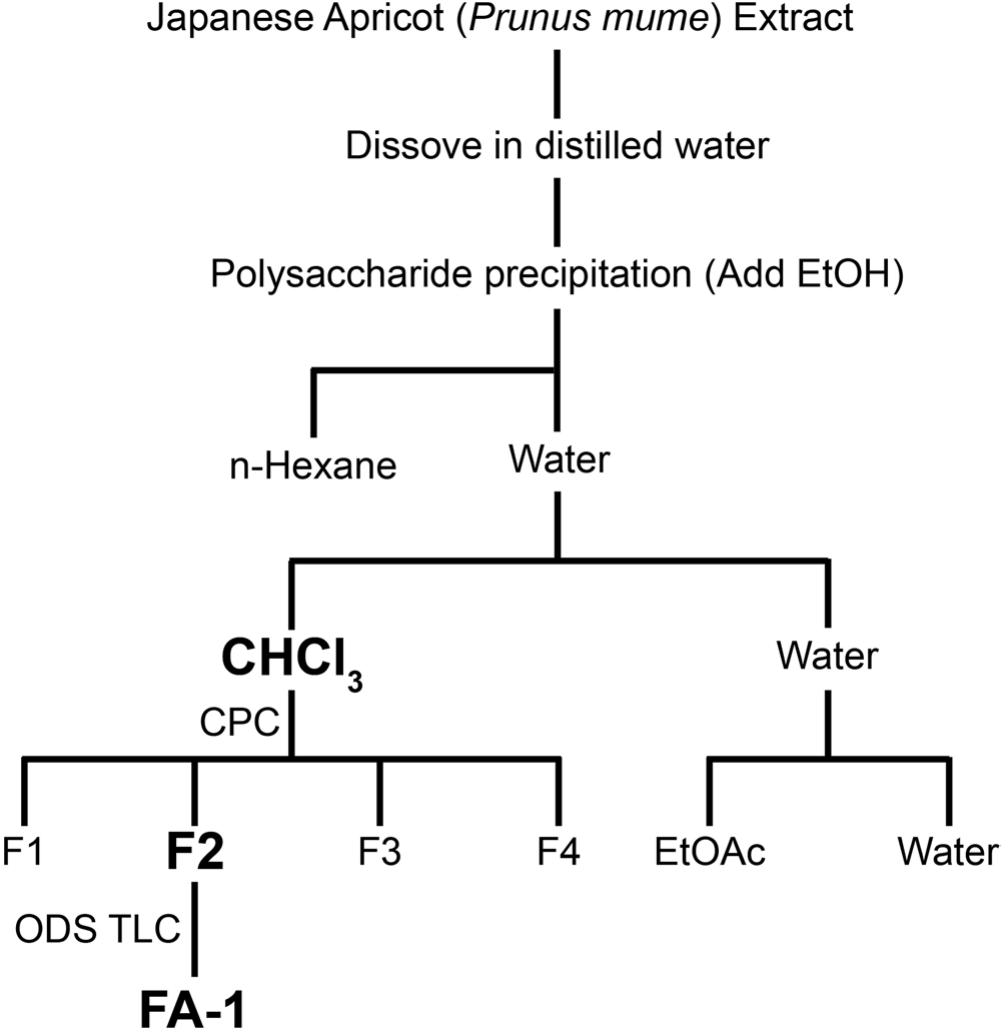
Purification scheme of FA-1 (5-hydroxymethyl-2-furaldehyde bis(5-formylfurfuryl) acetal) from JAE.

Concentrated JAE (250 g) was dissolved in distilled water (1 L), and ethanol (2 L) was added to remove polysaccharide precipitation. Thereafter, *n*-hexane was added and we performed water:chloroform extraction, obtaining the chloroform fraction (9.26 g) from the water fraction. Next four subfractions (F1, F2, F3 and F4) were prepared from the chloroform fraction using an ODS column. These four subfractions were screened for their ability to protect cells *in vitro* from CSE induced-cytotoxicity (data not shown). The F2 fraction conferred the highest protection against CSE induced-cytotoxicity (Supplementary Fig. S1). Therefore, centrifugal partition chromatography (CPC) was performed using two phases that were composed of *n*-hexane:ethyl acetate (EtOAc):methanol (MeOH):water = 3:7:5:6 (v/v) for direct isolation of active compounds from the chloroform fraction. The eluate (F2, 815.25 mg) from the CPC was monitored at 280 nm by UV^22^. To purify active compound, FA-1 (18.53 mg) from the F2 fraction was successively isolated and purified using preparative ODS thin layer chromatography (TLC) using 50% and 60% MeOH (Fig. 2) as the solvent.

**Figure 3:**
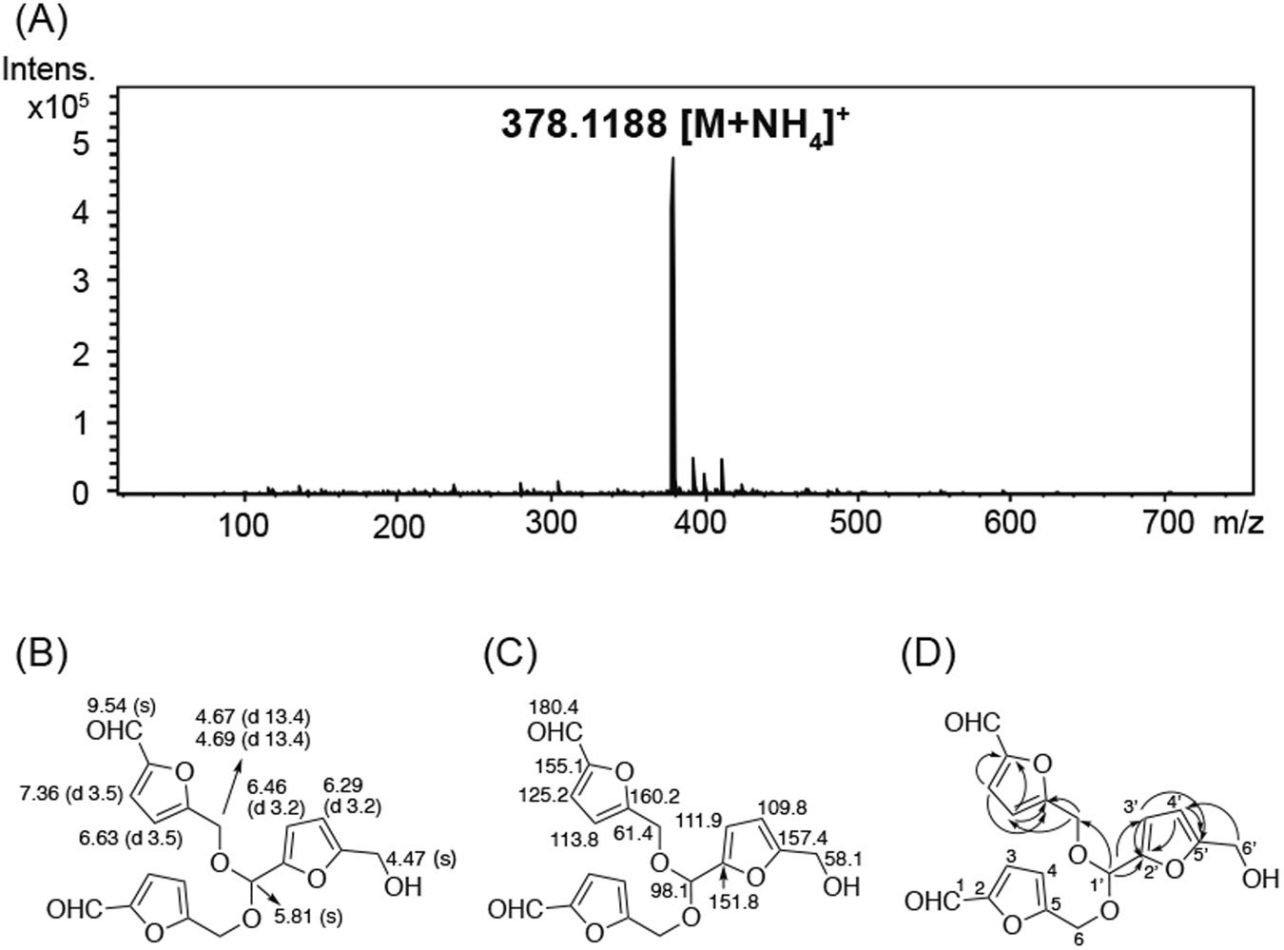
Determination of structure of FA-1 (5-hydroxymethyl-2-furaldehyde bis (5-formylfurfuryl) acetal). ESI-TOFMS data (**A**), ^1^H NMR data (chemical shifts in ppm and *J*_H,H_ values in Hz) (**B**), ^13^C NMR data (chemical shifts in ppm), and HMBC correlations (**D**) of FA-1.

FA-1 confers effective protection against CSE-induced cytotoxicity, so we sought to discover the structure of the FA-1 compound. The NMR data of FA-1 is shown in Fig. 3. ^1^H NMR, ^13^C NMR spectra, and the key HMBC correlations of FA-1 are illustrated in Fig. 3. FA-1 has the molecular formula C_18_H_16_O_8_ ([M + NH_4_]^+^ *m/z* 378.1188 calcd for C_18_H_20_NO_8_^+^ 378.1183 ESI-TOFMS) (Fig. 3A). The structure of this compound was elucidated as a trimer of 5-hydroxymethyl-2-furaldehyde based on their NMR spectra (^1^H NMR, ^13^C NMR, COSY, HSQC, and HMBC) (Supplementary Fig. S2–9). On the ^1^H NMR spectrum, two sets of similar signals of which the ratio of the integration values are almost 2:1 were shown. Both sets of these signals were assigned as the 5-hydroxy-2-furaldehyde skeleton on the basis of a conjugated aldehyde signal (δ_C_ 178.1, δ_H_ 9.54, s), two sets of olefinic methine protons coupling with each other with *J* values 3.2 and 3.5 Hz, two pairs of olefinic quaternary carbons (δ_C_ 151.8/157.4 and 155.1/160.2), and nonequivalent (δ_H_ 4.67, 4.69) and equivalent (δ_H_ 4.47) hydroxylmethyl proton signals. In addition, one acetal signal (δ_C_ 98.1, δ_H_ 5.81) was HMBC correlated with both of the furan C2 and nonequivalent hydroxymethyl signals. Together with other key HMBC correlations shown in Fig. 3D, the structure of this active compound was suggested to be 5-hydroxymethyl-2-furaldehyde bis (5-formylfurfuryl) acetal and was named FA-1. The assignment of ^1^H and ^13^C NMR signals are shown in Fig. 3B,C. NMR spectra are in Supplementary Fig. S2. To our best knowledge, this compound has never been reported from a natural source so far, although a patent containing this compound as a taste-improving agent can be found (WO 2008044784 A1 20080417).

### FA-1 increases ALDH and WRN proteins in a dose dependent manner and protects against CSE-induced cytotoxicity in immortalized human bronchial epithelial cells

**Figure 4:**
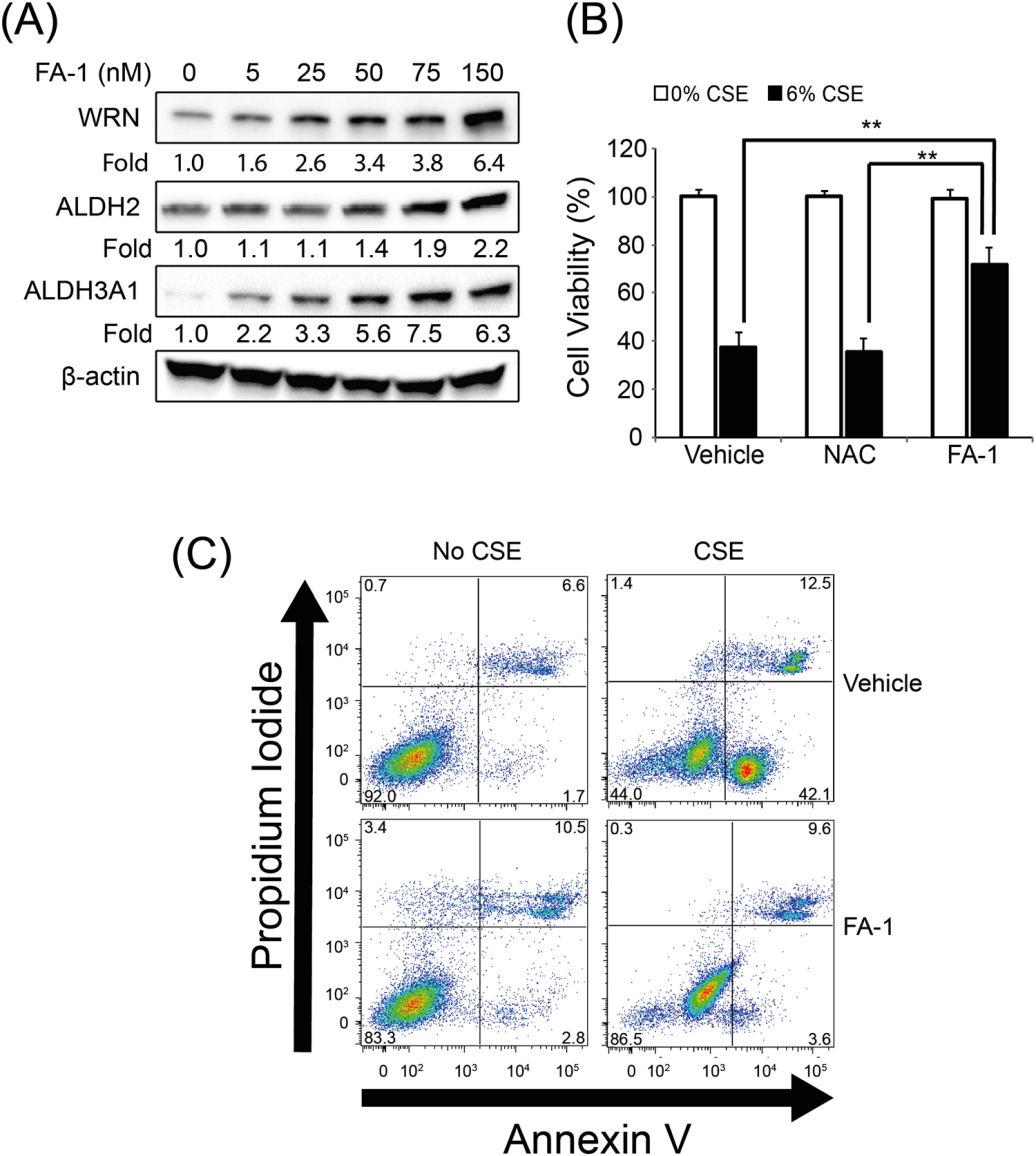
FA-1 increases ALDH and WRN proteins in a dose dependent manner and protects against cigarette smoke-induced cytotoxicity (MTT and flow cytometry) in immortalized human bronchial epithelial cells. (**A**) HBEC2 cells were cultured after treatment with various concentrations of the FA-1 (5, 25, 50, 75, and 150 nM in DMSO (dimethyl sulfoxide)) or the vehicle (Corresponding to 1.0% volume of DMSO). (**B**) HBEC2 cells were cultured with NAC (2 mM), FA-1 (150 nM), and vehicle in the presence or absence of 0 and 6% CSE for 24 h and assayed for viability. Data are expressed as mean ± SEM (**p < 0.01) cytotoxicity in cultured immortalized human bronchial epithelial cells; (**C**) Cell death was analyzed by annexin V and propidium iodide (PI) staining 24 h after CSE exposure. Representative flow cytometry data are shown.

To determine the effect of FA-1 on ALDH2, ALDH3A1, and WRN protein *in vitro*, HBEC2 cells were cultured after treatment with various concentrations of the FA-1 (5, 25, 50, 75, and 150 nM in DMSO (dimethyl sulfoxide)) or the vehicle (Corresponding to 1.0% volume of DMSO). FA-1 increases ALDH2, ADLH3A1, and WRN protein expression in a dose dependent manner (Fig. 4A). These data suggest that FA-1 enhances ALDH2, ALDH3A1 and WRN protein in HBEC2 cells and the effect of FA-1 was same as JAE. Thus, we decided and used 150 nM concentration of FA-1 for the next experiment. Furthermore, to determine the effects of FA-1 on CSE-induced cytotoxicity, HBEC2 cells were cultured with 2 mM of N-acetyl-L-cysteine (NAC), a thiol antioxidant or 150 nM of FA-1 in vehicle in the presence or absence of 6% CSE for 24 h. In this study, 2 mM of NAC did not confer protection against 6% CSE-induced cytotoxicity in HBEC2. However, FA-1 protected against 6% CSE-induced cytotoxicity according to the MTT assay (Fig. 4B). We further determined the effects of FA-1 on CSE-induced apoptosis using flow cytometry with both PI and Annexin V staining. FA-1 attenuates CSE-induced apoptosis (Fig. 4C). These results indicate that 150 nM of FA-1 increases ALDH and WRN protein and protects against CSE-induced apoptosis in HBEC2 cells.

### FA-1 attenuates CSE-induced oxidative damage and DNA damage in immortalized human bronchial epithelial cells

**Figure 5:**
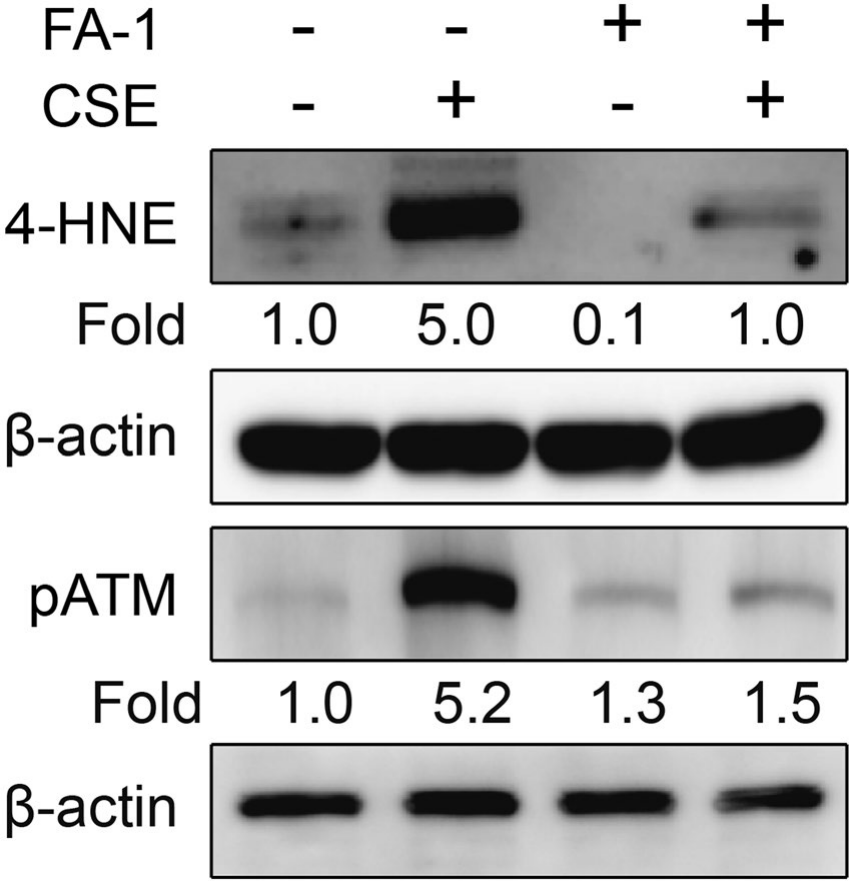
FA-1 attenuates cigarette smoke-induced oxidative damage and DNA damage in HBEC2 cells. HBEC2 cells were cultured with FA-1 (150 nM) in the presence or absence of 2% CSE for 24 h and immunoblot analysis was performed for 4-HNE and phosphorylation of ATM.

CS exposure is known to increase protein adducts of 4-HNE, a marker of oxidative stress^13^ and DNA damage as evidenced by an increase in phosphorylation of ATM^17,23^. To determine the effects of FA-1 on CS-induced oxidative stress and DNA damage, HBEC2 cells were cultured with or without CSE in the presence or absence of 150 nM FA-1 for 24 h. We found that FA-1 attenuates accumulation of 4-HNE protein adducts and phosphorylation of ATM in CSE-exposed HBEC2 cells (Fig. 5). These data indicate that FA-1 protects against CSE-induced oxidative stress and DNA damage in cultured HBEC2 cells.

### FA-1 increases ALDH2 and WRN protein and attenuates CSE-induced downregulation of WRN protein in normal human epidermal keratinocytes

**Figure 6:**
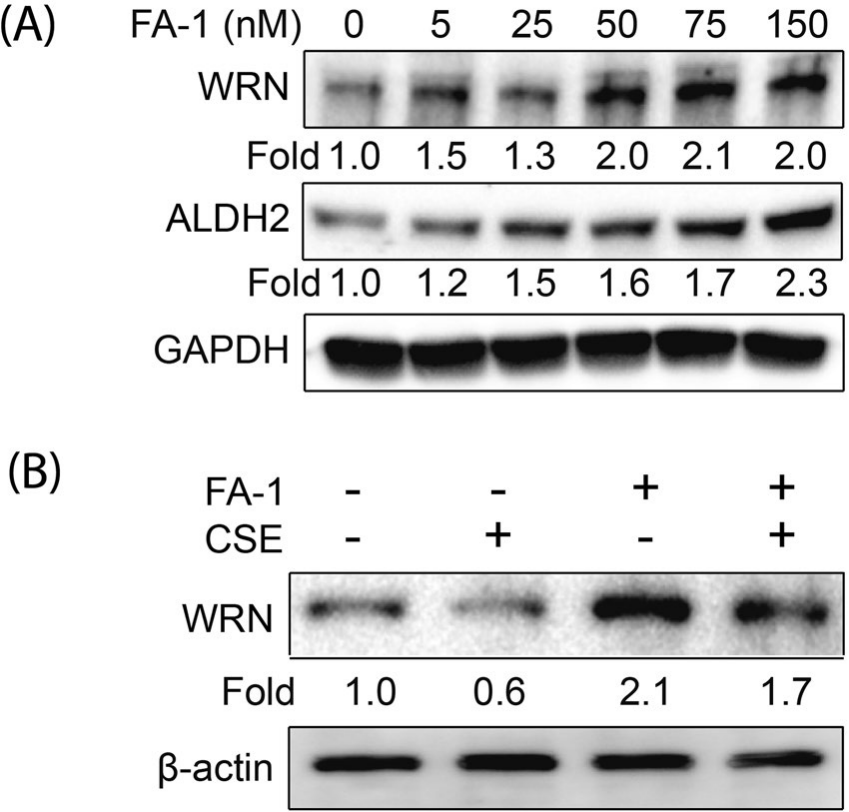
FA-1 increases ALDH2 and WRN proteins in a dose dependent manner and attenuates cigarette smoke-induced downregulation of WRN protein in normal human epidermal keratinocytes. (**A**) NHEK cells were cultured after treatment with various concentrations of the FA-1 (5, 25, 50, 75, and 150 nM in DMSO (dimethyl sulfoxide)) or the vehicle (Corresponding to 1.0% volume of DMSO). (**B**) NHEK cells were cultured with FA-1 (150 nM) and vehicle in the presence or absence of 2% CSE for 24 h.

To determine the effect of FA-1 on ALDH2 and WRN protein *in vitro*, NHEK cells were cultured after treatment various concentrations of the FA-1 (5, 25, 50, 75, and 150 nM in DMSO (dimethyl sulfoxide)) or the vehicle (Corresponding to 1.0% volume of DMSO). FA-1 enhanced ALDH2 and WRN protein expression in a dose dependent manner in NHEK cells (Fig. 6A). Due to the production of low ALDH3A1 protein in NHEK cells, ALDH3A1 was not detected. These data suggest that FA-1 protects against CSE-induced cytotoxicity and enhances ALDH2 and WRN protein in HBEC2 cells and the effect of FA-1 was same as JAE.

To determine the effects of FA-1 on CSE-induced downregulation of WRN protein, we cultured HBEC2 cells with FA-1 (150 nM) or the vehicle in the presence or absence of 1.5% CSE. FA-1 attenuated CS-induced downregulation of WRN protein in cultured HBEC2 cells (Fig. 6B). These data indicate that FA-1 preserve WRN protein expression and provides resistance to CS-induced downregulation.

### FA-1 protects against CSE-induced cytotoxicity and DNA damage in normal human epidermal keratinocytes

**Figure 7:**
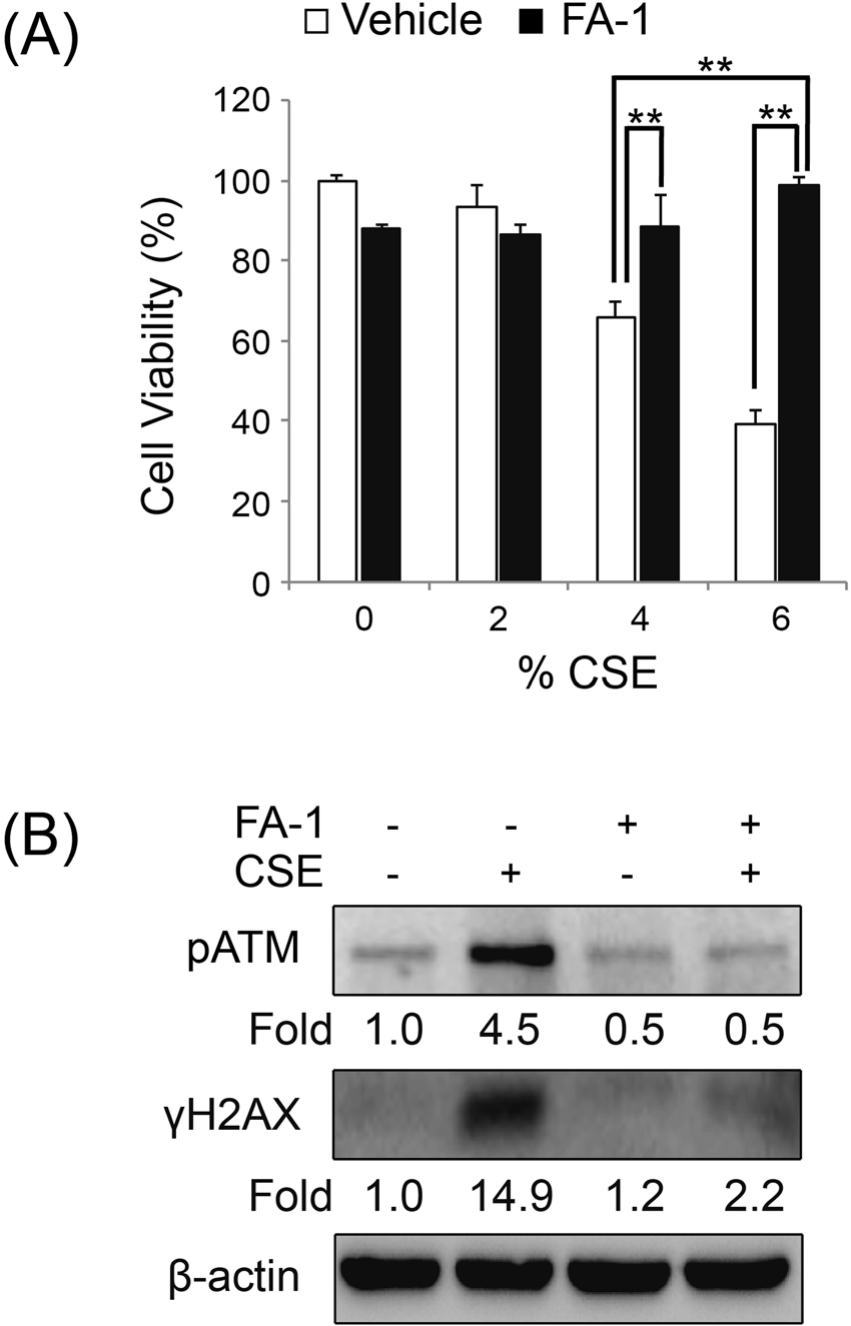
FA-1 protects against cigarette smoke-induced cytotoxicity and DNA damage in normal human epidermal keratinocytes. (**A**) NHEK MTT (3-(4,5-dimethythiazol-2-yl)-2,5-diphenyl tetrazolium bromide)-based cell viability after treatment with FA-1 (150 nM in DMSO (dimethyl sulfoxide)) in the presence or absence of 0, 2, 4, and 6% CSE for 24 h. Data are expressed as mean ± SEM (**p < 0.01) cytotoxicity in cultured NHEK; (**B**) NHEK cells were treated with 150 nM of FA-1 in the presence or absence of 2% CSE for 24 h. Immunoblot analysis was performed for phosphorylation of ATM and H2AX.

To determine the effects of FA-1 against CSE-induced cytotoxicity, NHEK cells were cultured with FA-1 (150 nM) or the vehicle. FA-1 protected against CSE-induced cytotoxicity according to the MTT assay (Fig. 7A). CSE exposure caused DNA damage as evidenced by an increase in phosphorylation of ATM and H2AX. To determine the effects of FA-1 on CSE-induced DNA damage, we cultured NHEK cells with FA-1 or the vehicle in the presence or absence of CSE. FA-1 decreased CSE-induced phosphorylation of ATM and H2AX (Fig. 7B). These data indicate that FA-1 protects against CSE-induced cytotoxicity and DNA damage in cultured NHEK cells.

## Discussion

This is the first report to demonstrate the protective effects of a novel compound, 5-hydroxymethyl-2-furaldehyde bis(5-formylfurfuryl) acetal, from concentrated JAE on CSE-induced cytotoxicity, DNA damage and oxidative stress in cultured HBEC2 and NHEK cells. We successfully isolated and determined the structure of 5-hydroxymethyl-2-furaldehyde bis(5-formylfurfuryl) acetal (which we named FA-1) from concentrated JAE, which has not been identified from a natural source to date. FA-1 protected against CSE-induced apoptotic cell death, DNA damage, and oxidative stress in cultured HBEC2 and NHEK cells. These protective effects of FA-1 were accompanied by increased protein expression of ALDH2, ALDH3A1, and WRN protein.

Japanese apricot contains various phytochemicals, such as polyphenolic and chlorogenic acid compounds^24^, and has antioxidant, anti-inflammatory, and innate immunity-enhancing biological effects^1–4^. JAE may prevent cardiovascular disease through an anti-oxidative effect on angiotensin II-induced ROS generation^6^. Triterpenoids in JAE have both anti-tumor and anti-inflammatory effects^7^. JAE has been reported to demonstrate an anti-inflammatory activity *in vitro*^5^. Finally, JAE has a skin-whitening effect by inhibiting melanin production via regulation of melanogenesis-associated protein expression in melanocytes^1^.

In this study, we isolated and identified the novel natural product FA-1, which confers effective protection against CSE-induced cytotoxicity *in vitro*, from concentrated JAE. The structure of FA-1 is based on a trimer of 5-hydroxymethyl-2-furaldehyde (HMF). HMF has been shown to have anti-oxidant and anti-inflammatory properties^25,26^. Furthermore, HMF significantly suppressed the hepatic levels of the pro-inflammatory response marker tumor necrosis factor-alpha (TNF-α) and interleukin-1β (IL-1β)^25^. Since the structure of FA-1 is based on a trimer of 5-HMF, for which there is a conjugate aldehyde, it is possible that the cytoin thiol (SH) of the enzyme and related proteins may be modified by the Michael reaction to form this compound. In the future, we will undertake to change the aldehyde conjugation of 5-HMF by organic synthesis method and determine whether the protective effects of FA-1 are changed.

In this study, we have shown that FA-1 protects against cigarette smoke-induced cytotoxicity and increases ALDH2 and 3A1 while preserving WRN protein in HBEC2 cells. Previous studies have demonstrated that JAE, from which FA-1 is extracted, induced the activity of alcohol metabolizing enzymes, such as alcohol dehydrogenase (ADH) and acetaldehyde dehydrogenase (ALDH)^27^. While ALDH1A3, ALDH2, ALDH3A2, ALDH3B1, ALDH5A1, ALDH19A1, ALDH16A1 and ALDH18A1 were significantly upregulated in CSE-exposed primary HBEC2 cells, no ALDH isozymes were downregulated by CSE. We previously reported that the effects of overexpression or suppression of ALDH3A1 on CSE-induced cytotoxicity and DNA damage (γH2AX) were evaluated in cultured, immortalized HBEC2 cells. Forced expression of ALDH3A1 attenuated cytotoxicity and downregulated γH2AX^14^. Furthermore, Patel and colleagues also showed that both gene and protein expression of ALDH3A1 were upregulated in airway epithelial cells of smokers relative to those of nonsmokers^11,28^. ALDH3A1-transfected cells are more resistant to cytotoxicity by various genotoxins, such as hydrogen peroxide, 4-HNE, mitomycin C, etoposide and ultraviolet light than are mock-transfected control cells^11–13^. Although lower intracellular concentration of 4-HNE promotes proliferation, antioxidant defense and compensatory mechanisms, higher concentration of 4-HNE triggers toxic pathways such as the induction of caspase enzymes, apoptotic cell death, and oxidative stress. Furthermore, 4-HNE has been linked in the pathology of Alzheimer’s disease, diabetes, cataract, and cancer^29,30^.

We further investigated the significant activity of FA-1 that attenuated CSE-induced apoptosis and DNA damage and augmented DNA repair in cultured HBEC2 and NHEK cells. FA-1 pretreatment significantly suppressed CSE-induced DNA damage and oxidative stress and also attenuated CSE-induced apoptosis. N-acetylcysteine (NAC) is an antioxidant drug with the potential to prevent lung injury^31–34^. Treatment of alveolar type II cells with NAC (0.5 μM) for 24 hours with low concentration of CSE (1.5% and 3%) significantly decreased apoptosis^34^. However, in this study, 2 mM of NAC did not confer such protection against 6% CSE-induced cytotoxicity in HBEC2, whereas 150 nM of FA-1 did protect against 6% CSE-induced cytotoxicity. We also reported that an antioxidant diet as compared to the regular diet significantly reduced neutrophilic inflammation and emphysema in mice exposed to a high concentration of cigarette smoke^35^. Additionally, we demonstrated that the brown algae derived compound, apo-9′-fucoxanthinone (Apo9F), confers robust protection against CSE-induced DNA damage and cytotoxicity in immortalized human bronchial epithelial cells^36^. Hsu *et al*. also reported Ginkgo biloba extract (EGb), which is a natural product, protected against CSE-induced oxidative stress-related apoptosis in human primary airway epithelial cells^37^. Furthermore, we reported that cigarette smoke induces cellular senescence and cell migration impairment via Werner’s syndrome protein down-regulation. Preservation of Werner’s syndrome protein may represent a potential therapeutic target for smoking-related diseases^16^. Hence, this observation is important because FA-1, which was isolated from concentrated JAE, augments multi-isozymes of ALDH2 and 3A1, and preserves WRN. Finally, FA-1 protects against CSE-induced DNA damage, oxidative stress, and apoptosis. Further studies will be required to determine the protection of FA-1 pretreatment on CS-induced emphysema and COPD using an *in vivo* system.

## Materials and Methods

### Chemicals, Reagents, and Antibodies

Chemicals were purchased from Sigma Aldrich (St. Louis, MO, USA), and proteinase and phosphatase inhibitor was from Thermo Scientific (Waltham, MA, USA). Antibodies were obtained from various sources: phosphorylation of anti-ATM (serine 1981), ALDH2, ALDH3A1 antibodies were from Abcam (Cambridge, UK); phosphorylation of anti-H2AX antibody was from Cell signaling (Beverly, MA); 4-HNE antibody was from R&D Systems (Minneapolis, MN); anti-β actin was from Sigma-Aldrich (St. Louis, MO).

### Isolation and Structural determination of JNYP

Concentrated Japanese apricot extract, *P. mume*, was provided from Fysee Inc. in South Korea and was immediately frozen and kept below −20 °C until use. FA-1 was isolated and purified as we previously described^22^. NMR spectra were recorded on an Agilent 600 MHz NMR spectrometer (Agilent Technologies, Santa Clara, CA USA) in 0.4 ml of CD_3_OD at 20 °C. Spectra were referenced to residual solvent signals with resonances at δ_H/C_ = 3.30/49.8 ppm (CD_3_OD). The signals were assigned based on the analyses of the COSY, HSQC, and HMBC spectra. MS analysis was recorded on a ESI-TOFMS (micrOTOF-Q II, Bruker, Billerica, MA, USA).

### Cigarette Smoke Extract Preparation

Research cigarettes (3R4F) from the University of Kentucky were purchased and used to make CSE solutions. CSE solutions were prepared as previously described^16^.

### Cell culture and Cell viability

Immortalized human bronchial epithelial cells (HBEC2) were provided by Drs Shay and Minna^38^, Southwestern Medical Center, Dallas, TX, and maintained as previously described^11^; normal human epidermal keratinocytes (NHEK cells; ATCC, Manassas, VA) were cultured in keratinocyte serum-free medium supplemented with 5 µg/L of human recombinant epidermal growth factor and 50 mg/L of bovine pituitary extract (Invitrogen) in plates coated with FNC coating mix (Athena ES). All cells were grown under standard incubator condition at 37 °C and with 5% CO_2_. Experiments were performed in 12-well and 6-well tissue culture plates at a starting cell density of 10 × 10^3^/cm^2^.

Cell viability was determined by measuring the reduction of 3-(4,5-dimethylthiazol-2-yl)-2,5-diphenyl tetrazolium bromide (MTT) as we previously described^39^. HBEC2 and NHEK cells were cultured in 12 well plates for 24 H and treated with 150 nM of FA-1 that was dissolved with DMSO (Sigma Aldrich) for 24 H. After treatment for 24 H, the cells were exposed to various concentrations (2%, 4%, and 6%) of CSE for 24 H. Absorbance was measured at 540 nm, and the relative cell viability of CSE-exposed cells was determined by comparing the vehicle control cells, unexposed to CSE.

### Flow Cytometric analysis of apoptotic cells

After treatment with FA-1 (150 nM) and exposure to CSE, cells were harvested by trypsinization. 10^5^ cells were stained in 1 x binding buffer (0.01 M HEPES, pH 7.4; 0.14 M NaCl; 0.25 mM CaCl_2_) using 10 µL of propidium Iodide (PI) and 5 µL of Annexin V-FITC (BioLegend, San Diego, CA, USA). The cells were incubated in the dark room for 15 min at room temperature and the percentage of FITC- and PI-positive cells were quantified using FACS Canto-II flow cytometer (BD Biosciences, San Jose, CA, USA) and were analyzed using Flowjo software (version 7.6.3; TreeStar, San Carlos, CA, USA).

### Immunoblot analysis

After treatment with FA-1 (150 nM) and exposure to CSE, we harvested cell lysate with 1X RIPA buffer (Sigma-Aldrich), including protease and phosphatase inhibitor (Thermo Fisher). Protein concentration was determined with BCA protein assay reagent (Thermo Fisher). Protein samples were separated with SDS-PAGE and transferred to polyvinylidene fluoride membrane. The membrane was blocked with 5% non-fat milk in TBS with 0.5% Tween-20, and incubated with primary and second antibodies, and visualized with enhanced chemiluminescence reagent (Advansta). The approximate positions (kDa) of prestained protein standard (Bio-Rad) are indicated on the right of the blots. Equivalent loading was verified by stripping the blot and reprobing with antibodies to β-actin. Relative quantification of signal intensity was determined using image Lab software (Bio-Rad, Hercules, CA, USA) and expressed as a relative densitometry.

### Statistical analysis

For all comparisons involving multiple treatment groups, one-way ANOVA was used to identify treatment effects. *p*-values, based on calculated contrasts, were used to assess the individual treatment effects. The results are expressed graphically as the mean ± SEM, and *p* < 0.05 was considered statistically significant.

## Electronic supplementary material

Supplementary information
