# Supplementary material for: A Novel Compound, “FA-1” Isolated from Prunus mume, Protects Human Bronchial Epithelial Cells and Keratinocytes from Cigarette Smoke Extract-Induced Damage
Source: Sci Rep. 2018 Jul 31;8:11504. doi: 10.1038/s41598-018-29701-2 (PMC6068145; doi:10.1038/s41598-018-29701-2)
Supplement: Supplementary file 1 — Supplementary information [file 41598_2018_29701_MOESM1_ESM.pdf]

Supplementary information

A Novel Compound, "FA-1" Isolated from *Prunus mume*, Protects Human Bronchial Epithelial Cells and Keratinocytes from Cigarette Smoke Extract-Induced Damage

Andrew J. Jang[1,7*], Ji-Hyeok Lee[2], Mari Yotsu-Yamashita[3], Joo-dong Park[4], Steve Kye[5], Raymond L. Benza[1], Michael J. Passineau[1], You-Jin Jeon[6], Toru Nyunoya[7]

[1]Cardiovascular Institute, Department of Medicine, Allegheny Health Network, Pittsburgh, PA 15212, USA.

[2]Lee Gil Ya Cancer and Diabetes Institute, 7-45, Songdodong, Yeonsugu, Incheon 406-840, Republic of Korea

[3]Graduate School of Agricultural Science, Tohoku University, 468-1 Aramaki-Aza-Aoba, Aoba-ku, Sendai, Miyagi, 980-0845, Japan

[4]Fysee Inc., 131, Angam-ro, Angseong-myeon, Chungju-si, Chungcheongbuk-do 27303, Republic of Korea

[5]Acerta Pharma, 2200 Bridge Parkway, Suite 101 Redwood City, CA 94065, USA

[6]Department of Marine Life Sciences, Jeju National University, Jeju 690-756, Republic of Korea

[7]Department of Medicine, University of Pittsburgh, Pittsburgh, PA 15213, USA

*Corresponding author. Tel.: +1-412-359-6153; fax: +1-412-359-4698.

Email addresses: junho.jang@ahn.org (Andrew Junho Jang, PhD)

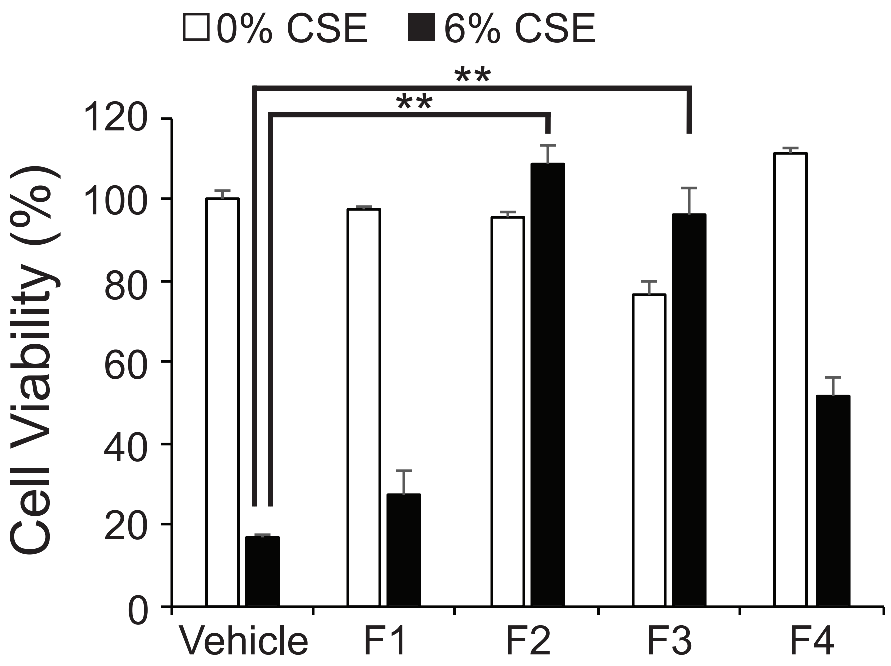

Figure S1. Cell viability of F1, 2, 3 and 4 from chloroform fraction on CSE-induced cytotoxicity.

2. NMR spectra of FA-1 (5-hydroxymethyl-2-furaldehyde bis(5-formylfurfuryl) acetal)

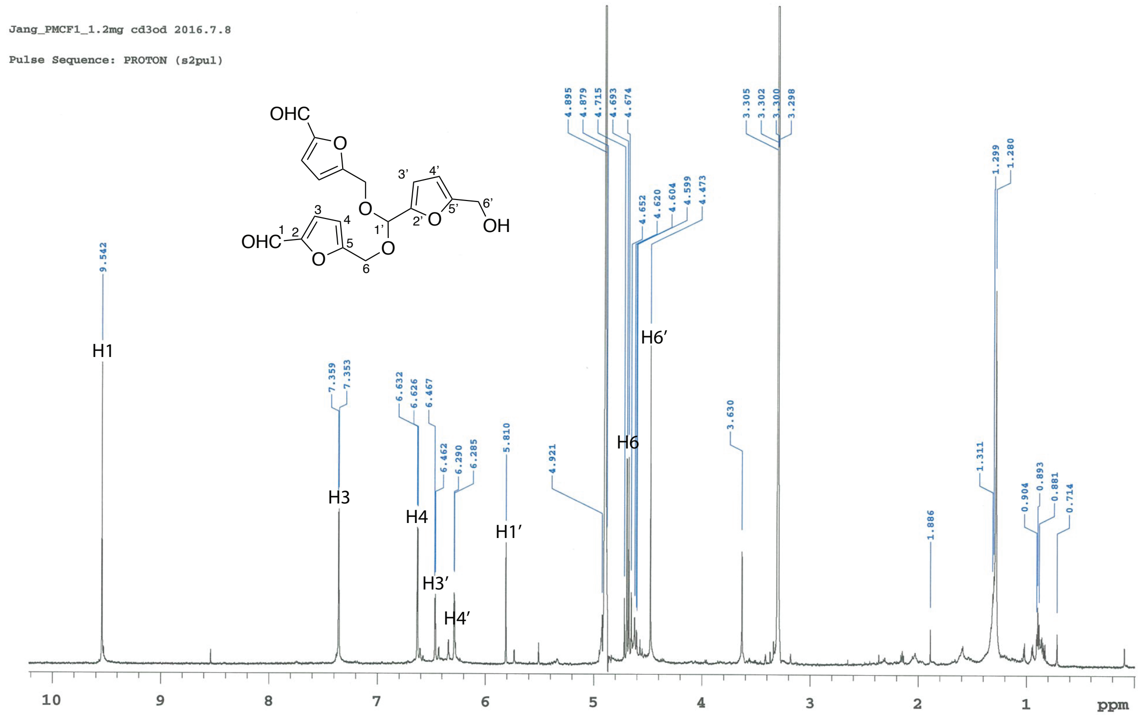

Figure S2. ^{1}H NMR spectrum (600 MHz, CD_3OD)

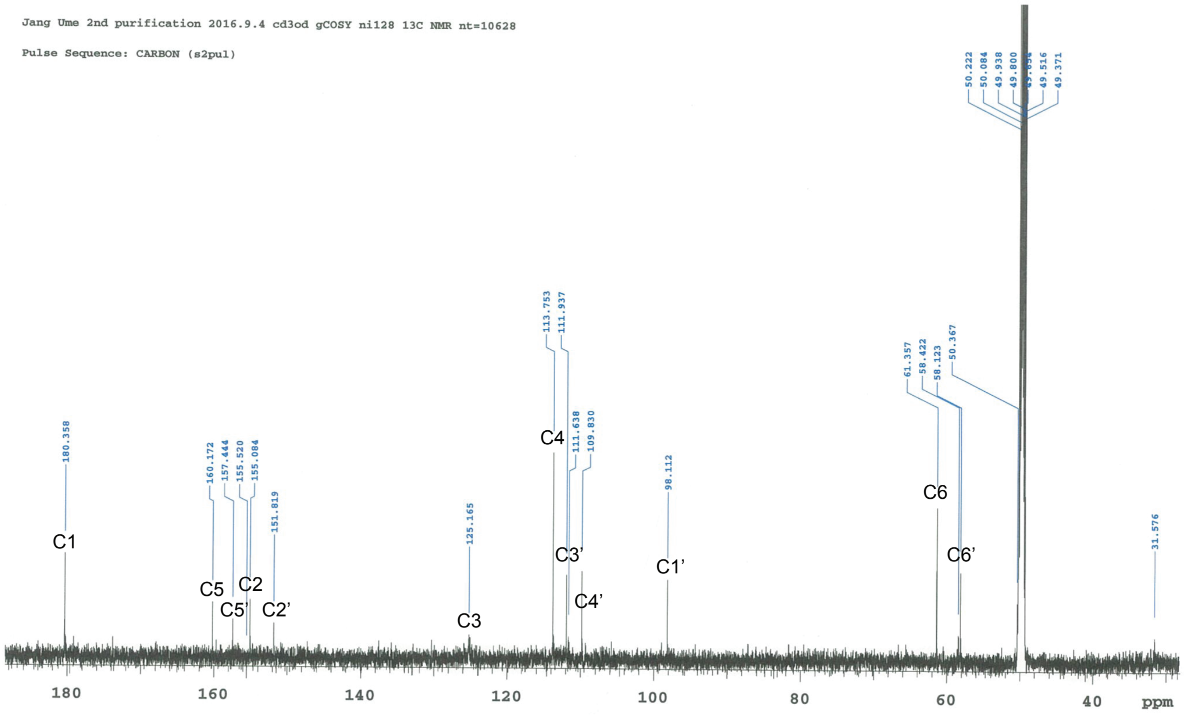

Figure S3. ^{13}C NMR spectrum (151 MHz, CD_3OD)

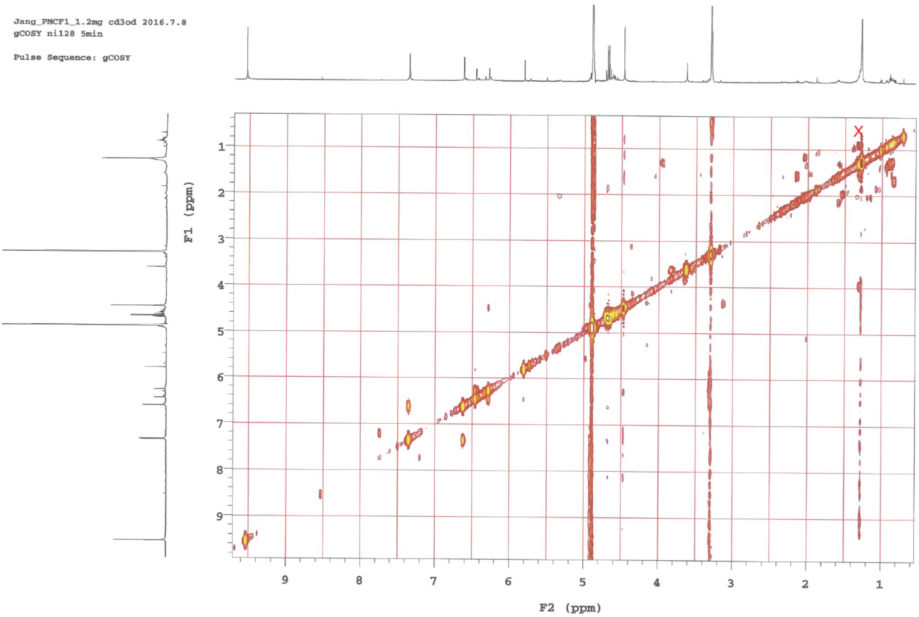

Figure S6. COSY spectrum (600 MHz, CD_3OD)

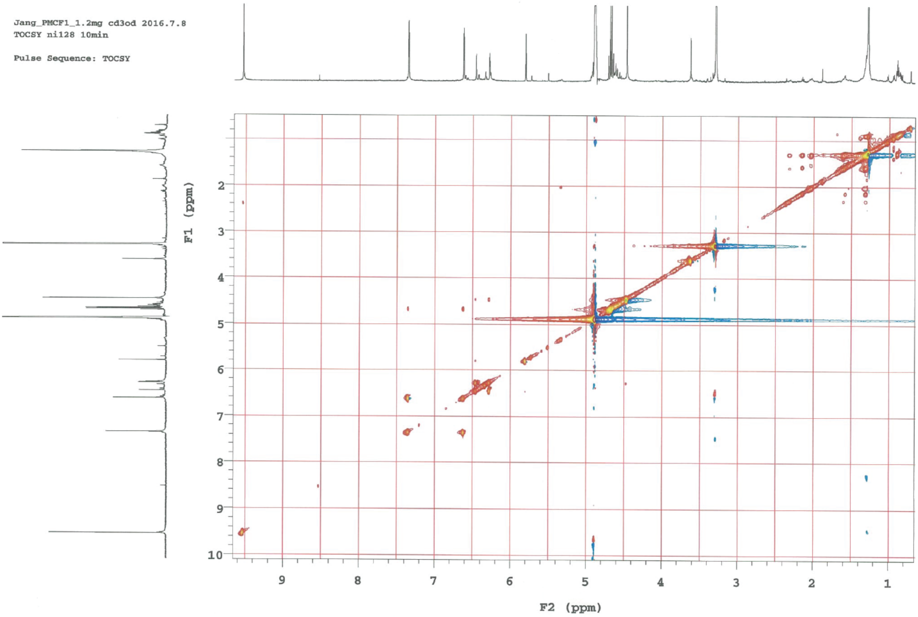

Figure S7. TOCSY spectrum spectrum (600 MHz, CD_3OD)

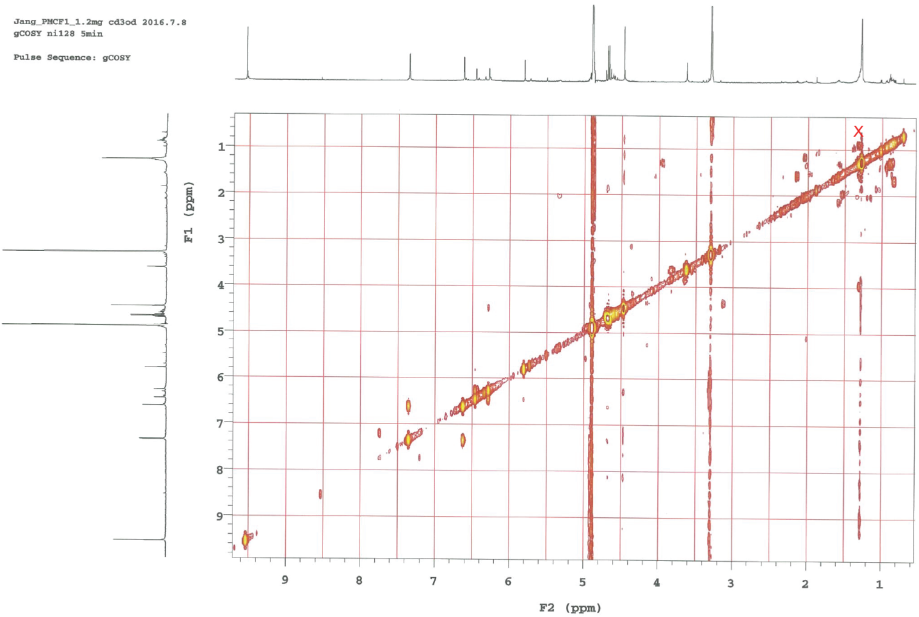

Figure S6. COSY spectrum (600 MHz, CD_3OD)

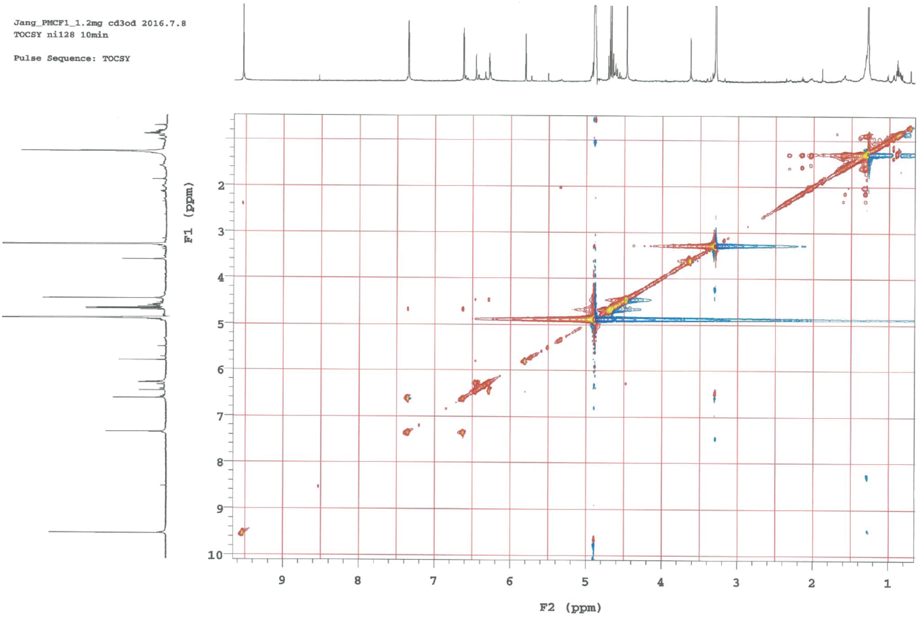

Figure S7. TOCSY spectrum spectrum (600 MHz, CD_3OD)

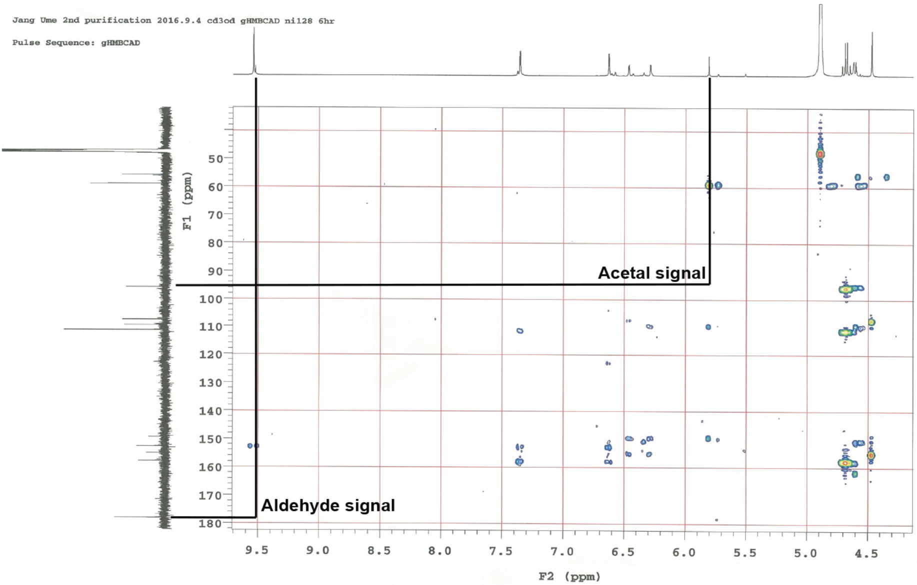

Figure S8. HMBC spectrum (600 MHz, CD_3OD)

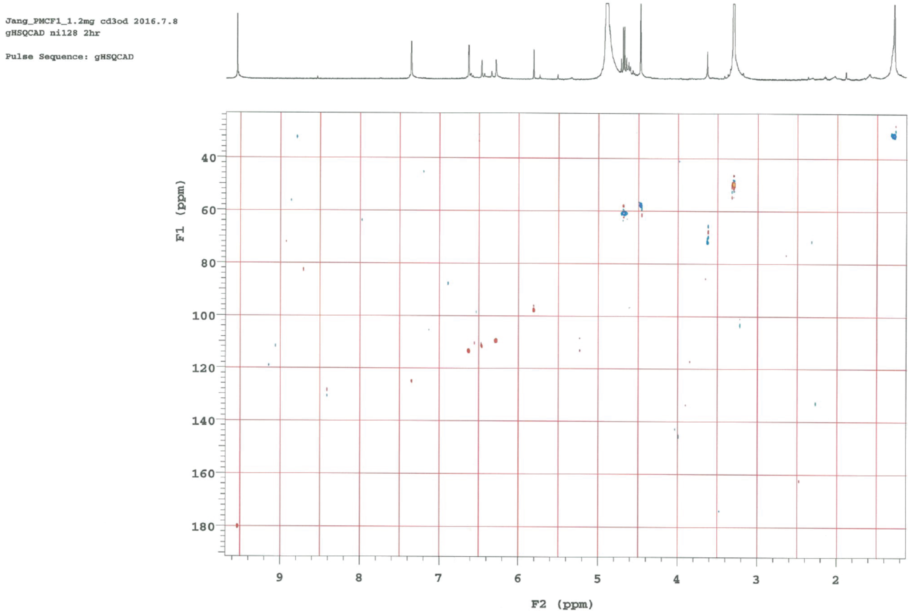

Figure S9. HSQC spectrum (600 MHz, CD_3OD)
